# Implant success and survival rates in daily dental practice: 5-year results of a non-interventional study using CAMLOG SCREW-LINE implants with or without platform-switching abutments

**DOI:** 10.1186/s40729-018-0145-3

**Published:** 2018-11-02

**Authors:** Sven Marcus Beschnidt, Claudio Cacaci, Kerem Dedeoglu, Detlef Hildebrand, Helfried Hulla, Gerhard Iglhaut, Gerald Krennmair, Markus Schlee, Paul Sipos, Andres Stricker, Karl-Ludwig Ackermann

**Affiliations:** 1 Private practice, Baden-Baden, Germany; 2 Private practice, Munich, Germany; 3 Private practice, Istanbul, Turkey; 4 Private practice, Berlin, Germany; 5 Private practice, Strass in Steiermark, Austria; 6 Private practice, Memmingen, Germany; 7 Department Oral and Maxillofacial Surgery/Plastic Surgery, University Hospital Freiburg, Center for Dental Medicine, Freiburg, Germany; 8 Private practice, Marchtrenk, Austria; 9 Private practice, Forchheim, Germany; 10 Department of Maxillofacial Surgery, Goethe University Frankfurt, Frankfurt, Germany; 11 Private practice, Amstelveen, Netherlands; 12 Private practice, Constance, Germany; 13 Private practice, Filderstadt, Germany

**Keywords:** Implant success, Implant survival, Dental implants, Patient satisfaction, Daily dental practice, Non-interventional study, Platform switching, Hard tissue, Soft tissue

## Abstract

**Background:**

The performance of dental implants in controlled clinical studies is often investigated in homogenous populations. Observational studies are necessary to evaluate the outcome of implant restorations placed in real-life situations, according to standard practice, and to assess the needs of the patients. The aim of this non-interventional study was to reveal the survival, success, and general performance of CAMLOG SCREW-LINE implants and their restorations in daily dental practice.

**Methods:**

Seventeen private practices across five countries participated in this prospective multicenter study. Patients received implants in the maxilla and mandible which were restored either with platform-matching or platform-switching abutments. Patients were followed-up for up to 5 years post-loading. Radiographs and clinical parameters were evaluated and patient satisfaction was evaluated.

**Results:**

From a total of 196 patients planned, 185 patients with 271 implants were restored with abutments and fulfilled the follow-up inclusion criteria. Three implant failures were recorded, resulting in a cumulative survival rate of 98.6% after 5 years post-loading. One persistent complication of peri-implantitis occurred. The soft tissue health remained stable, and the papilla height improved after loading. At 5-year follow-up, the mean crestal bone loss was − 0.28 ± 0.60 mm; over 99% of patients reported satisfaction with the restoration as excellent or good.

**Conclusions:**

Implants placed and restored with both platform-matching and platform-switching abutments in daily dental private practice achieved excellent clinical outcomes with highly satisfied patients after 5 years of function, confirming the results obtained in well-controlled clinical trials.

## Background

Success and survival rates of endosseous implants are well-documented in a number of controlled clinical trials and systematic reviews [1–3]. Generally, controlled trials evaluate endosseous implants in specific clinical situations; thus, the patient population is subjected to rigorous inclusion criteria and follow-up. Accordingly, controlled clinical trials do not reflect the real-life situation in private practice. Numerous factors including experienced clinicians, specialized clinics, restricted inclusion and exclusion criteria, specific indications, and increased time spent during follow-up, may affect or even bias the results, outcomes, or reported implant success and survival rates [4]. Consequently, there is an increasing trend in assimilating and reporting real-life data [4] allowing for the evaluation and assessment of dental implants in daily practice. An observational, non-interventional study in a non-homogeneous population better reflects daily practice than a controlled clinical study.

Various studies have reported on success and survival of endosseous implants in private practice settings. In a 5-year prospective observational study on 590 patients, Cochran et al. [5] evaluated 990 implants placed under routine private practice conditions. Very high cumulative survival and success rates were achieved after 3 years (> 99%) and 5 years (97%) of loading. These results were found to be comparable with the rates of survival and success of the same sand-blasted, large grit, acid-etched (SLA) implants achieved in a controlled, prospective, multicenter clinical study (Cochran et al. 2002 as cited in [5]).

Nevertheless, observational studies performed in private practice are not without their flaws; studies have shown that patients may be poorly motivated to attend follow-up appointments [6, 7], and results from various studies imply that, in non-controlled clinical settings, follow-up attendance may drop when patient satisfaction is high [6–8]. In contrast, regular follow-up appointment attendance is integral to the study design in controlled clinical trials; therefore, the patient’s obligated attendance to follow-up appointments may mask their natural behavior when satisfied.

In the present study, CAMLOG SCREW-LINE implants with the Promote plus surface (sandblasted and acid-etched surface) were used. These implants in combination with platform-matching abutments have been shown to have high long-term success rates ranging from 97.8 to 100% at 5-year to 10-year follow-up [9–13]. They can be restored with either platform-matching or platform-switching abutments with the difference that platform-switching abutments have a narrower diameter than the implant, leading to an implant-abutment mismatch. The effects of platform switching on hard tissue outcomes are well-studied, with there being a tendency to better outcomes with respect to crestal bone loss with platform switching [14–23]. Regarding the CAMLOG SCREW-LINE implants, the effect of platform switching and platform matching was evaluated in a randomized controlled clinical trial (RCT) [21, 23]. At 1-year follow-up, implant success rates were 97.3% and 100%, and at 3 years, implant survival was 97.3% and 97.1%, for platform-switching and platform-matching implants respectively. Platform-switching implants showed a positive effect on marginal bone loss already at 1-year follow-up, and significantly less marginal bone loss was reported with the platform-switching versus platform-matching technique at 3 years (0.28 ± 0.56 mm vs. 0.68 ± 0.64, respectively; *p* = 0.002).

To understand the performance of the CAMLOG SCREW-LINE implants used with platform-switching and platform-matching abutments outside of a controlled clinical environment, we conducted a prospective, non-interventional study in private practice. The primary objective was to provide data for a life table analysis on the performance of the implants in private practice, to show the probability of survival and success of the dental implants after a follow-up time of 5 years post-loading. Secondary objectives were to evaluate patient satisfaction through the assessment of appearance, ability to chew, ability to taste, comfort, general satisfaction, and fit. The outcomes were also reported for platform-switching and platform-matching subgroups.

## Methods

### Study design

This was a prospective multicenter non- interventional study to assess implant success and survival rates in daily dental practices using the CAMLOG SCREW-LINE implants (CAMLOG Biotechnologies AG, Basel, Switzerland) used with or without platform-switching abutments. Patients were enrolled over a period of 2 years from October 2008 to September 2010 from 17 sites across five countries (Austria *n* = 2, Germany *n* = 10, Spain *n* = 2, the Netherlands *n* = 2, and Turkey *n* = 1). All patients gave their signed informed consent for participation in this study. The study was performed in accordance with the declaration of Helsinki, and institutional review board approval was obtained from the respective local review boards of the participating countries. The reporting of this study conforms to the STROBE statement [24].

The primary outcome of this study was the implant survival and success rates at 1, 3, and 5 years post-loading. The secondary outcomes were patient satisfaction as indicated by the assessment of the patient’s ability to chew, ability to taste, comfort, appearance and fit of restoration, and general satisfaction.

### Population

Male and female patients ≥ 18 years of age with sufficient bone at the implant site to achieve primary stability were included in this study. It was expected that the patients would return to the treatment center for prosthetic restoration and routine follow-up appointments at 1, 3, and 5 years post-loading. If socket preservation were to be performed, a minimum of 6 months must have elapsed before surgery. In such cases, this would be documented on the case report form. Patients were excluded if they had any contraindications to the package insert for the dental implant system, if primary stability at the implant insertion was not achieved, or if any bone graft and/or guided bone regeneration procedure was required. The treatment indications were single or multiple tooth replacement in the maxilla or mandible without the use of simultaneous augmentation or membrane, of which the implants were to be restored with either fixed single crown or fixed partial denture restorations.

### Treatment procedure

Patients were to be treated according to standard practice for implant procedures applicable in the countries participating in the study. Implants used in this study were CAMLOG SCREW-LINE implants (K-Line) with diameters of 3.8 mm, 4.3 mm, 5.0 mm (or 6.0 mm), and lengths of 9 mm, 11 mm, and 13 mm. Both platform-matching and platform-switching abutments could be used. The protocol allowed freedom of choice, and the investigators selected the best option for the patients’ indication. Implants were placed following normal treatment protocols of the participating site and were inserted following one-stage or two-stage surgery decided upon clinical need. Implants were restored after a healing period of at least 6 weeks post-surgery in bone class I–III and 12 weeks in bone class IV [25]. During surgery, the bone quality, crestal ridge width and height, and primary stability of the implant were documented.

### Follow-up

The post-surgical examination took place between 1 and 2 weeks post-surgery according to the standard practice. At this time, patient complaints and adverse events were recorded. Patients underwent suture removal and were instructed in oral hygiene and plaque removal. Follow-up visits were scheduled according to standard practice and according to the surgical protocol. Patients undergoing two-stage surgery attended a re-entry surgery for placement of the healing abutment; otherwise, patients attended the clinic for abutment placement, provisional prosthesis placement, and definitive prosthesis placement as per individual treatment plan. Follow-up appointments then occurred at 6 months, 1, 2, 3,4, and 5 years post prosthetic installation. Standard maintenance care like check-ups for dental hygiene was performed as required.

### Assessments

Throughout the study, only radiographs consistent with standard implant procedures were taken. Bone level changes were assessed based on available and evaluable standardized periapical radiographs with a film-holder using parallel-technique or panoramic radiographs (depending on the standard in the study centers). Baseline was defined as the time of the first prosthetic installation (loading). Each investigator performed their own measurements on either digital or analog radiographs, as available. In order to achieve standardized measurements, all analog radiographs were digitized and measurements were performed on all radiographs with the free-available software ImageJ 1.50i by an experienced independent person and subsequently validated by the investigators. All periapical and panoramic radiographs were individually calibrated (distance of three threads) to account for the distortion of the pictures. The distance from the implant shoulder to the first visible bone contact at the mesial and distal aspect of the implants was measured. The measurements at the mesial and distal site were averaged to obtain the bone level per implant. The changes in the bone level were calculated over several intervals: from loading to 5 years post-loading and at yearly intervals starting from 1 year post-loading to evaluate the success criteria. Bone quality [26] was assessed during the surgery (D1 to D4). Clinical parameters to assess the soft tissue health, including Modified Plaque Index (MPI), Papilla Index, Sulcus Bleeding Index (SBI), and pocket probing depth (PPD) (if measured), were recorded during abutment placement, during placement of the definitive prosthesis, and at each subsequent follow-up visit. The MPI and SBI were measured according to the criteria described by Mombelli [27]. The presence of the mesial and the distal papilla was evaluated according to the Jemt papilla score [28]. PPD, if routinely measured, was evaluated as a change in probing depths at 1, 3, and 5 years, compared with baseline. MPI, SBI, and PPD were determined on the buccal, lingual, distal, and mesial sites on each implant; the mean value of the scores was taken to provide the assessment of the implant.

The primary stability of the implant was assessed during surgery. Implant success and survival were evaluated in the group of implants restored with abutments [5, 29] at both placements of the provisional and definitive prostheses and at each follow-up visit thereafter. Implants were deemed successful in accordance with the criteria for implant success laid down by Albrektsson et al. [30]. Implants were successful if there was less than 0.2 mm bone loss annually after the first year of loading, if they were clinically immobile, if there was no peri-implant radiolucency, and if there was no persistent and/or irreversible pain, infection, neuropathies, or paresthesia. During the course of the study, the criterion of bone loss by Albrektsson et al. was scrutinized, and the scientific relevance was not considered to be suitable anymore [30, 31]; therefore, implant success was assessed post hoc, according to Buser et al. [29], that is, there was no persistent and/or irreversible signs or symptoms such as pain, infection, neuropathies, or paresthesia, no peri-implant infection with suppuration, no mobility, and no continuous radiolucency around the implant. Radiological evaluation for radiolucency and bone loss was measurable only with available evaluable radiographs; radiographs for some patients were missing or not evaluable. In the case that no complications were reported by the clinician, and the patients reported being satisfied according to the set criteria, then radiographs were not necessary and the implant was deemed successful.

The patients rated their satisfaction regarding the ability to chew, to taste, their comfort, appearance and fit of restoration, and general satisfaction on a categorical scale (very unsatisfied, unsatisfied, middle, satisfied, very satisfied) via a questionnaire at each visit beginning from loading [17, 22].

### Safety

Adverse events were recorded on an adverse event form and reported as non-treatment associated or treatment-associated events.

### Statistical methods

A minimum of 200 patients were planned to be included in the study. Analyses were performed on the per protocol population. In addition, to assess the correlation of implant success with anatomical and surgical parameters, analyses of the subgroups “platform matching” and “platform switching” (based on abutment type) were performed. Implant success and survival rates were calculated using a life table analysis 1 year after baseline and yearly thereafter. To test for significant differences for repeated measurements, the Wilcoxon signed-rank test was used, and to assess for significant differences between the subgroups, the Kruskal-Wallis test was used. *p* values of less than *p* < 0.05 were deemed significant. Changes in the crestal bone were quantitatively evaluated through the standardized measurements of the radiographs. Any non-standardized radiographs allowed for qualitative analysis only. Standardized measurements on radiographs for calculating bone level changes were done with the freely available software ImageJ 1.50i (https://imagej.nih.gov/ij/). Descriptive statistics were performed with IBM SPSS Statistics for Windows V24.0 (IBM Corp., Armonk, NY, USA).

## Results

### Patient demographics

**Fig. 1:**
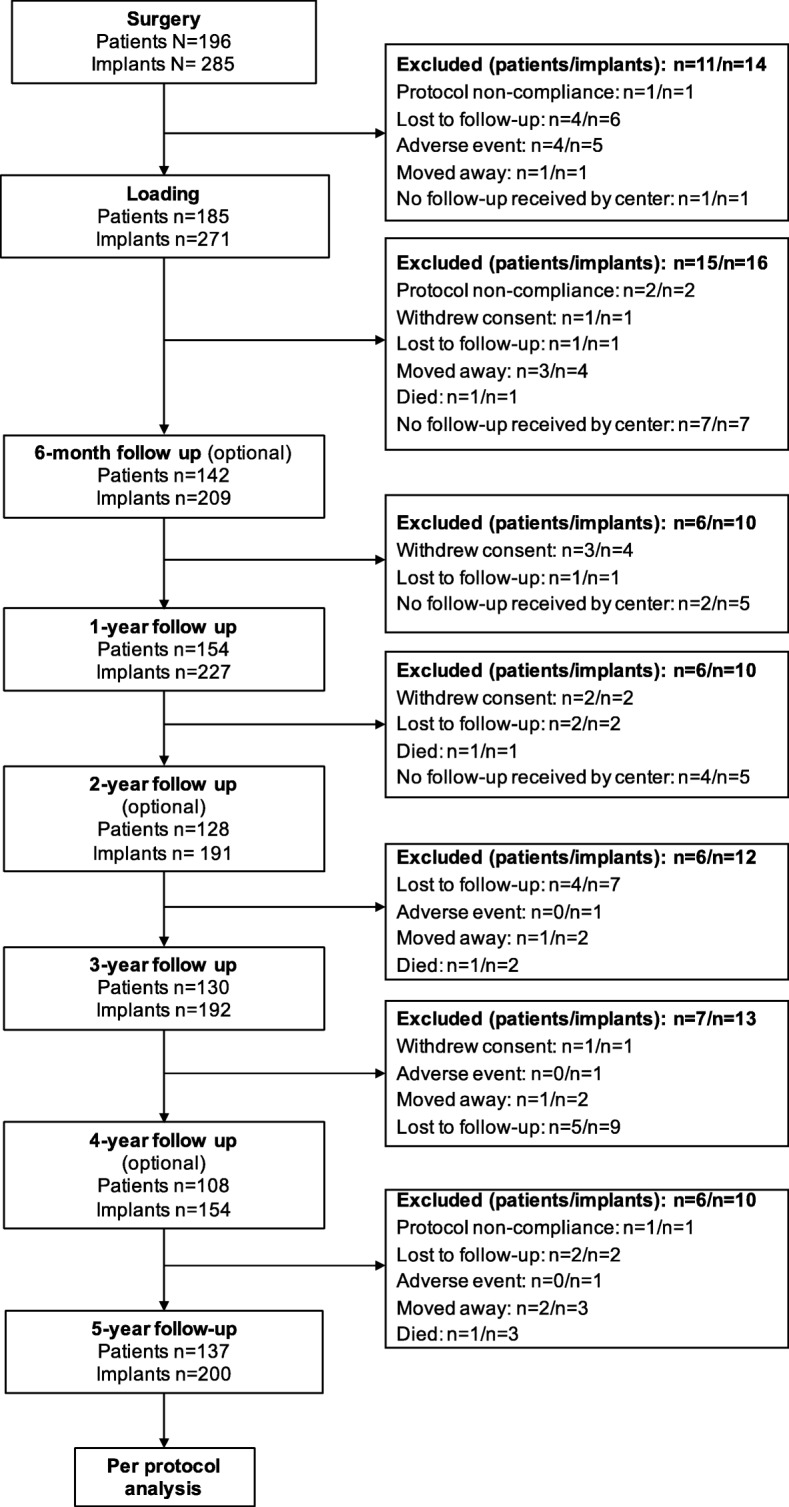
Study flow diagram: follow up status and reasons for not completing the study; six-month, 2-year and 4-year follow up was optional

In total, 196 patients from 17 centers met the inclusion criteria for this study and were included in the per-protocol analysis. In total, 285 implants were placed (Table 1). At the 5-year follow-up, data were available for the 137 patients who completed the study (Fig. 1). Patient demographic data is presented in Tables 2 and 3.

**Table 1:** Table of study centers

Investigator*	City/country	Number of patients included	Number of implants included
Dr. Helfried Hulla	Strass in Steiermark, Austria	10	15
Prof. DDr. Gerald Krennmair	Marchtrenk, Austria	10	20
Dr. S. Marcus Beschnidt (PI)	Baden-Baden, Germany	8	12
Dr. Karl-Ludwig Ackermann	Filderstadt, Germany	14	18
Dr. Thomas Barth	Leipzig, Germany	15	28
Dr. Claudio Cacaci	Munich, Germany	11	14
Dr. Christian Hammächer	Aachen, Germany	10	13
Dr. Detlef Hildebrand	Berlin, Germany	16	30
PD Dr. Gerhard Iglhaut	Memmingen, Germany	4	5
PD Dr. Dr. Markus Schlee	Forchheim, Germany	18	22
Dr. Dr. Manfred Wolf	Leinfelden-Echterdingen, Germany	14	17
PD Dr. Dr. Andres Stricker	Constance, Germany	11	18
Dr. Juan Manuel Vadillo	Madrid, Spain	12	17
Dr. Fernando Loscos Morató	Zaragoza, Spain	15	22
Dr. Gert de Lange / Dr. Paul Sipos	Amstelveen, Netherlands	15	18
Dr. Chris van Lith	Hoorn, Netherlands	4	4
Dr. Kerem Dedeoglu	Istanbul, Turkey	9	12

*All in private practice

**Table 2:** Patient demographics

	Overall	Subgroup*	
		Platform switching	Platform matching
Patients, *n* (%)	196 (100)	144	41
Sex, *n* (%)			
Male	87 (44.4)	62 (43.1)	19 (46.3)
Female	109 (55.6)	82 (56.9)	22 (53.7)
Age, years			
Mean (SD)	51.5 (14.2)	53.1 (14.4)	47.4 (12.9)
Range	17.9–82.1	17.9–82.1	19.3–78.5
Pre-implant bone surgeries, *n*			
Autogenous bone grafting	31	n/a	n/a
Socket preservation	1	n/a	n/a
Others	16	n/a	n/a
Pre-implant soft tissue surgeries, *n*			
Palatal soft tissue graft	15	n/a	n/a
Other	1	n/a	n/a
Bone quality, %			
D1: mainly homogenous bone	12.8	8.9	22.4
D2: compact bone thick	42.2	42.4	41.8
D3: compact thin/cancellous good density	42.2	45.3	35.8
D4: compact thin/cancellous low density	2.8	3.4	0.0
Reasons for tooth loss, *n* (%)			
Caries	53 (19.3)	36 (18.4)	13 (19.4)
Endodontic	96 (35.0)	67 (34.2)	24 (35.8)
Fracture	35 (12.8)	32 (16.3)	3 (4.5)
Periodontal	46 (16.8)	33 (16.8)	13 (19.4)
Endodontic and periodontal	3 (1.1)	3 (1.5)	0 (0.0)
Caries and periodontal	4 (1.5)	4 (2.0)	0 (0.0)
Endodontic and fracture	2 (0.7)	1 (0.5)	1 (1.5)
Others	35 (12.8)	20 (10.2)	13 (19.4)
Missing	11 (3.9)		
Smoking status, *n* (%)			
Non-smoker	166 (86.5)	121 (85.2)	37 (90.2)
Mild smoker (≤ 10/day)	18 (9.4)	13 (9.2)	4 (9.8)
Heavy smoker (> 10/day)	8 (4.2)	8 (5.6)	0 (0.0)
General health status, *n* (%)			
ASA P1	161 (85.6)	118 (85.5)	34 (82.9)
ASA P2	26 (13.8)	20 (14.5)	6 (14.6)
ASA P3	1 (0.5)	0 (0.0)	1 (2.4)

*Eleven patients with 14 implants were not loaded/restored with abutments due to early implant failures or because the patients were lost to follow-up (Fig. 1)

**Table 3:** Patient demographics with respect to implants

		Overall	Subgroup*			
			Platform switching		Platform matching	
Total Implants, *n*		285	203*		68*	
Number of implants placed per patient, *n* (%)						
1		125 (63.8)	97 (67.4)		20 (48.8)	
2		56 (28.6)	37 (25.7)		16 (39.0)	
3		12 (6.1)	7 (4.9)		5 (12.2)	
4		3 (1.5)	3 (2.1)		0 (0.0)	
Implant position distribution, *n*						
Maxilla						
17		3	2		1	
16		16	10		4	
15		14	8		5	
14		9	8		0	
13		1	1		0	
12		11	10		1	
11		8	7		1	
21		11	10		1	
22		7	6		1	
23		8	8		0	
24		11	8		2	
25		11	7		3	
26		15	12		2	
27		2	1		1	
Mandible						
47		12	8		4	
46		44	31		10	
45		13	9		3	
44		9	7		2	
43		1	0		1	
42		1	1		0	
41		0	0		0	
31		1	1		0	
32		0	0		0	
33		0	0		0	
34		4	1		3	
35		14	9		4	
36		47	33		13	
37		12	5		6	
Diameter	Length of implant				Total	
	9 mm	11 mm	13 mm	16 mm	Total, *n*	Total, %
3.3 mm	0	1	0	0	1	0.4
3.8 mm	10	29	54	1	94	33.0
4.3 mm	15	43	50	2	110	38.6
5.0 mm	7	35	36	1	79	27.7
6.0 mm	0	1	0	0	1	0.4
Total *n*	32	109	140	4	285	100.0

*Eleven patients with 14 implants were not loaded/restored with abutments due to early implant failures or because the patients were lost to follow-up (Fig. 1)

### Implant success

Implant success was reported according to the criteria for implant success laid down by Albrektsson et al. [30], as well as that by Buser et al. [29]. According to Albrektsson et al., there were three implant failures post-loading and three implants which did not meet the success criteria due to bone loss (*n* = 2) and peri-implantitis (*n* = 1). According to Buser et al., there were three implant failures post-loading and one implant which did not meet the success criteria due to peri-implantitis (*n* = 1) (Table 4). The three implants which were late failures were lost at 2 years post-loading due to important bone loss and at 3.6 years and at 4.6 years post-loading (all platform switching). Additional five implants were lost before loading as a result of no osseointegration (early failures) and therefore were not considered for the analysis.

**Table 4:** Life table analysis showing the cumulative success rate according to Albrektsson et al. and Buser et al.

Interval (months)	Implants in interval	According to Albrektsson et al.			According to Buser et al.		
		Implants withdrawn during interval	Failures during interval	Cumulative success rate (%)	Implants withdrawn during interval	Failures during interval	Cumulative success rate (%)
Loading – 12	271	27	0	100	27	0	100
12–24	244	6	0	100	6	0	100
24–36	238	17	1	99.6	17	1	99.6
36–48	220	11	3	98.2	13	1	99.1
48–60	206	50	2	97.1	50	2	98.0
60–72	154	94	0	97.1	94	0	98.0
72–84	60	48	0	97.1	48	0	98.0
> 84	12	12	0	97.1	12	0	98.0

The cumulative success rates did not differ according to both criteria at 1-year follow-up or at 3-year follow-up, being 100% and 99.6%, respectively. However, at 5-year follow-up, the success rate according to Buser et al. was higher at 98.0% than that according to Albrektsson et al. at 97.1%. The sub-group analysis revealed that the success rate for platform-matching implants was 100% at 1-year and at 3-year and 96.2% at 5-year follow-up according to Albrektsson et al. and 100% at each follow-up according to Buser et al. Conversely, for platform-switching implants the success rate was 100% at 1-year follow-up, 99.4% at 3-year follow-up, and 97.4% at 5-year follow-up, according to both criteria.

### Implant survival

The cumulative survival rate was 100% at 1-year follow-up, 99.6% at 3-year follow-up, and 98.6% at 5-year follow-up. All three late failures were in the platform-switching subgroup.

### Clinical parameters/soft tissue parameters

#### Plaque index

**Fig. 2:**
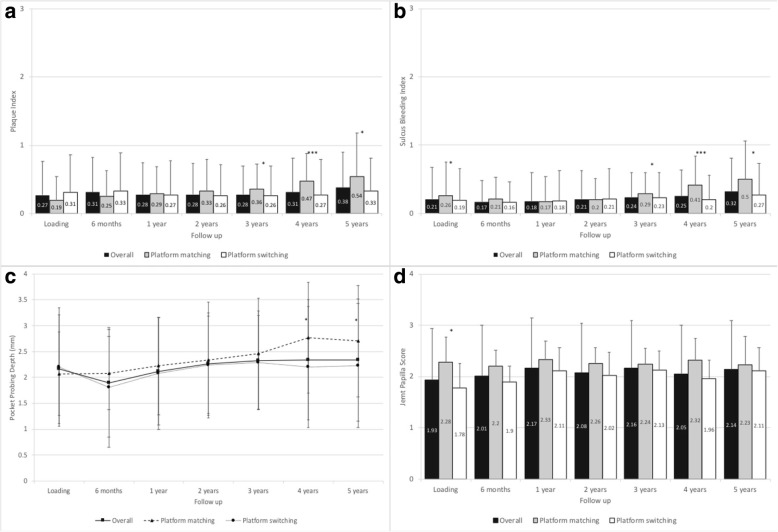
Clinical parameters and soft tissue parameters. **a** Modified plaque index. Error bars indicate standard deviation. * = *p* ≤ 0.05, *** = *p* ≤ 0.001. **b** Sulcus bleeding index. Error bars indicate standard deviation. * = *p* ≤ 0.05, *** = *p* ≤ 0.001. **c** Pocket probing depth. The asterisk represents statistically significant differences (* = *p* ≤ 0.05) observed between subgroups. **d** Jemt papilla score. The asterisk represents statistically significant differences (* = *p* ≤ 0.05) observed between subgroups

Mean modified plaque indices were very low at below 0.5 for all but one measurement throughout the course of the study (Fig. 2a). At loading, the overall MPI was 0.27 ± 0.49 slightly increasing to 0.38 ± 0.52 at 5-year follow-up; the increase in MPI from loading to 5-year follow-up was statistically significant (*p* < 0.001). The MPI for the platform-switching subgroup was significantly lower than that for platform-matching subgroup at 3-year (*p* = 0.025), 4-year (*p* = 0.001), and 5-year (*p* = 0.028) follow-up.

#### Sulcus bleeding index

At loading, the overall SBI was 0.21 ± 0.47, remaining very low throughout the study and slightly increasing to 0.32 ± 0.49 at 5-year follow-up (Fig. 2b). The increase in SBI from loading to 5-year follow-up was statistically significant (*p* < 0.001). At 3-year follow-up, the SBI was significantly higher in the platform-matching subgroup than in the platform-switching subgroup (0.29 vs. 0.23; *p* = 0.039); this difference increased at 4-year and 5-year follow-up (0.41 vs. 0.20; *p* = 0.001 and 0.50 vs. 0.27; *p* = 0.004, respectively).

#### Pocket probing depth

At loading, the PPD was 2.16 ± 1.05 mm; it decreased to 1.89 ± 1.04 mm at 6 months post-loading, increasing to 2.12 ± 1.04 mm at 1-year follow-up (Fig. 2c). From this point onward, the PPD increased to 2.34 ± 1.18 mm at 5-year follow-up. The increase in mean PPD from loading to 5-year follow-up was statistically significant (*p* = 0.032). The mean PPD for the platform-switching subgroup was significantly lower than that for the platform-matching subgroup at 4-year (2.20 mm vs. 2.77 mm; *p* = 0.012) and 5-year follow-up (2.23 mm vs. 2.70 mm; *p* = 0.011).

#### Jemt papilla score

At loading, the Jemt papilla score was 1.93 ± 1.01, significantly increasing to 2.14 ± 0.95 at 5-year follow-up (*p* = 0.023) (Fig. 2d). For the platform-switching subgroup, a significant difference was observed between baseline and 5-year follow-up (*p* < 0.001); however, no significant difference was observed for the platform-matching group over the same time period. Furthermore, at loading, the Jemt papilla score was significantly lower for the platform-switching subgroup than for the platform-matching subgroup (1.78 vs. 2.28; *p* = 0.009).

#### Bone level changes

**Fig. 3:**
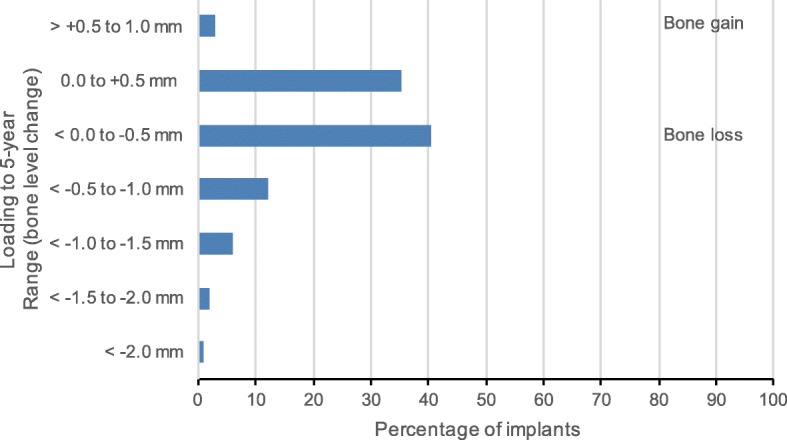
Bone level changes from loading to 5-year follow up

Evaluable radiographs were available from 13 participating sites: eleven sites with periapical and two sites with orthopantographic radiographs. At loading and at 5-year follow-up, respectively, 148 and 119 evaluable radiographs were available. The mean bone level change from loading (baseline) to 5-year follow-up was (mean ± SD) − 0.28 ± 0.60 mm. No significant differences in the mean bone level change from loading to 5-year follow-up were observed between the platform-switching and platform-matching subgroups (− 0.32 ± 0.60 mm vs. − 0.13 ± 0.29 mm). From loading to 5-year follow-up, no bone loss or even bone gain was observed in 38% of evaluable implants. Figure 3 shows the frequency distribution of bone level changes from loading to 5-year follow-up for all implants.

#### Patient satisfaction

**Fig. 4:**
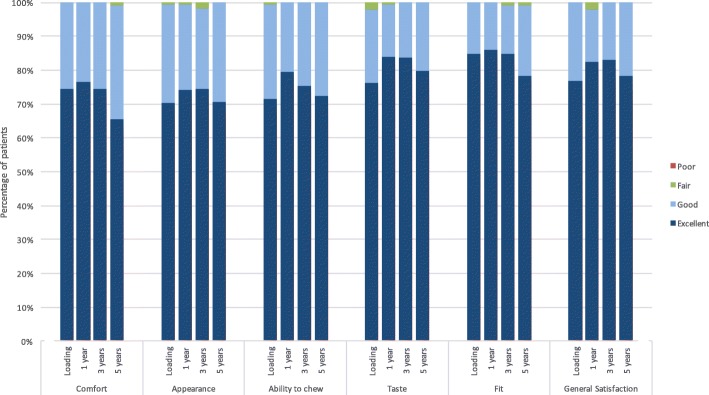
Patient satisfaction throughout the study

Patient satisfaction was reported as excellent by over 60% of all patients for each category at each time point during the course of the study, with almost all remaining patients reporting good outcomes (Fig. 4). No more than three patients reported an outcome of fair for any category at any time point, and no patients reported an outcome of poor for any category at any time points. No differences were observed between the platform-switching and platform-matching subgroups for any category at any time point (data not shown).

#### Prosthetic complications

With regard to prosthetic complications, there were two cases of ceramic chipping in two patients; the restorations were corrected and no further complications were seen. There were three cases of crown loosening which resolved after re-cementing the crowns, and there were two cases of abutment screw loosening leading to crown mobility, which resolved after screw tightening.

## Discussion and conclusions

This large, multicenter study provides real-life long-term data on 285 implants placed in 196 patients. The results show that the placement of CAMLOG SCREW-LINE implants with platform-matching or platform-switching abutments results in high survival and success in the long term. The overall success rate for implants was 97.1% at 5-year post-loading, and 97.4% and 96.2% for implants with platform-switching and platform-matching abutments, respectively, according to Albrektsson et al. [30]; the overall survival rate was 98.6%. For comparability to other studies, the success rates were assessed post hoc according to Buser et al. [29], revealing a 5-year overall success rate of 98.0%, and 100% and 97.4% for implants with platform-matching and platform-switching abutments, respectively.

These results compare positively with the results achieved for the CAMLOG SCREW-LINE implants in an RCT [23]. Here, the 3-year success rates—according to Buser et al. [29]—were 97.3% for platform-switching and 97.1% for platform-matching implants. In contrast, the present study achieved better 3-year success rates—according to Buser et al. [29]—for both platform-matching (100%) and platform-switching (99.4%) implants. Other private practice studies achieved similar results to our study, with success rates at 3 years of 93.5% for SLActive implants [4] and 99.12% and 97.58% at 3 and 5 years, respectively, for comparable SLA surface implants [5]. These studies [4, 5] also applied the success criteria, according to Buser et al. [29], namely absence of pain, infection, neuropathies or paresthesia, peri-implant infection with suppuration, mobility, and continuous radiolucency around the implant. Slight differences in success rates are seen with the two criteria [29, 30]. In our study, the success rates are lower at 5-year follow-up, according to Albrektsson et al., because bone level changes were measured to fulfill the first criterion (< 0.2 mm bone loss annually after the first year of loading). At 3-year follow-up, bone loss was noted in one patient (reclassified as peri-implantitis at the 4-year follow-up) and an important bone loss (due to poor oral hygiene and bruxism; two implants) in a patient with psychosocial issues who could not be treated during the study. Such a patient would not have been included in an RCT. Consequently, three implants were lost based on the bone loss criterion. Being able to measure bone level changes is also dependent on the availability of evaluable radiographs. In our study, these were taken as per standard clinical protocol using the available equipment, which may differ to that available in a university clinic, a setting commonly found in controlled clinical studies. Thus, some radiographs were not digitized and were difficult to read. Also, if the protocol does not stipulate radiography, then the natural behaviors of patients in private practice are revealed. Some patients refused radiographs, other patients were followed up by referring dentists, and radiographs were not exchanged. Additionally, if radiographs are routinely acquired, the clinician is still reliant on follow-up attendance. Accordingly, the success rates measured in the present study should be assessed collectively. Other studies not assessing bone level changes may report higher success rates than those achieved if bone level changes were evaluated [4, 5].

Other factors need to be considered when reporting success [32]. Papaspyridakos et al. reported a relationship between the number of success criteria and the success rate: the higher the number of success criteria, the lower the reported success rate [32]. Also, the common criterion of bone loss being < 2.0 mm during the first year of function, followed by < 0.2 mm annually thereafter, may no longer be suitable, particularly with new implant systems, such as platform-switching implants, which lead to minimal crestal bone remodeling (Prosper et al. and Trammell et al. cited in [32]). Over the 5-year study period, we report < 2.0 mm bone level change for all implants, 0.1–0.5 mm for 40%, and no bone loss or bone gain for 38% of all implants. Additionally, bone loss was 0.32 ± 0.66 mm and 0.13 ± 0.29 mm for the platform-switching and platform-matching subgroups. Of note, in this study, the platform-matching and platform-switching groups were very unbalanced (67 vs. 206 implants) because the decision to choose abutment type was the clinician’s choice according to the clinical situation. Furthermore, very few radiographs were available for the platform-matching subgroup; thus, differences between the two subgroups are not conclusive. Nevertheless, the minimal crestal bone loss of 0.32 mm observed for platform-switching implants is comparable with the data reported in other studies on platform-switching implants [17, 23]. The bone gain of 0.12 ± 0.42 mm at 1-year follow-up [17] and of 0.16 ± 0.53 mm at 3 years follow-up [23] have been reported. In these studies, the outer geometry of the implant was comparable; however, Rocha et al. [23] used implants of the same kind while Moergel et al. [17] used implants with a conical connection.

The importance of the vertical soft tissue thickness has recently been reported [33, 34]. Platform-switching implants placed in thick tissues led to the preservation of the crestal bone level, while this was not observed in thin mucosal tissues. These studies were not yet published in the planning phase and initiation of the present study. Accordingly, pocket probing depth measurements were performed rather than vertical soft tissue thickness. These measurements may be biased; it is thought that the probe may stop at the horizontal shift instead of the pocket depth, yet, to our knowledge, there is no reference supporting this. In daily practice, probing was sometimes not performed if the implants showed no pathological findings. On the one hand, the variety of bone level changes in this study may be explained by different vertical soft tissue thicknesses, but cannot be validated due to these missing data. On the other hand, there are multiple confounding factors influencing the change in bone level, such as the size of the platform (mismatch), occlusal loading, and the microgap.

Additional to the standard success criteria, patient-reported outcomes are important factors when evaluating an implant system [32]. In our study, if the patient was satisfied, no further radiographs were taken, and the implant was deemed successful. Furthermore, in some success criteria, overall patient satisfaction should be good or excellent for the treatment to be successful (Levi et al. cited in Papaspyridakos et al. [32]). Our study reveals an exceptionally high level of patient satisfaction. The majority of patients reported excellent outcomes for all measured categories at each time point throughout the study, with most remaining patients reporting good outcomes (Fig. 4). No patient reported a poor outcome, and a maximum of three patients at any given time reported fair outcomes. The parameters assessed by patients are closely related to soft tissue outcomes, which reflect oral hygiene and soft tissue health. The soft tissue parameters assessed in our study were MPI, SBI, PPD, and Jemt papilla score. For MPI, a statistically significant increase was observed from loading to the 5-year follow-up; however, the MPI at 5-year follow-up was, at 0.38 ± 0.52, still very low, with 0 equaling no detection of plaque and 1 equaling plaque only detectable after running a probe across the smooth marginal surface of the implant [27]. Similarly, the SBI remained very low throughout the study, despite a significant increase from loading to 5-year follow-up. At 5-year follow-up, the overall SBI was 0.32 ± 0.49, reflective of no bleeding given that 0 equals no bleeding and 1 equals isolated bleeding spots visible [27]. The PPD initially decreased within the first 6 months from which point it significantly increased to 2.34 ± 1.18 mm at 5-year follow-up. Nevertheless, the measured mean PPD still reflects the norm for conventionally placed implants, which at 2–4 mm is indicative of healthy tissues [35]. The same trend was observed for the Jemt papilla score [28], which significantly increased from loading to 5-year follow-up (2.14 ± 0.95). The ideal papilla score of 3 [28]corresponds to the optimal soft tissue contours; thus, the scores achieved in our study are close to the ideal. Although we observed some significant differences in these parameters between the platform-switching and platform-matching subgroups at 5 years, these are not clinically significant.

Our study should be particularly noted for its ability to recall patients for follow-up appointments. Patient attendance at follow-up appointments in trials performed in private practice can be troublesome [4, 6–8], and the inability to obtain full data from all patients at the later stages of a study may limit the interpretation of the final results. We obtained data for the 70% of patients completing the study at 5 years; this minimizes the limitations in the interpretation of results seen in comparable studies [5–8]. Although this study was performed in private practice, the investigators are very experienced in implantology and of good standing and understand the importance of follow-up and maintenance of good oral health. We observed a maximum of only five patients with poor oral hygiene at any given time (data not shown); additionally, the three late implant failures were in two patients with peri-implantitis or poor hygiene. The appearance of poorer oral hygiene later in the study also appears to correspond with the drop in follow-up attendance, which again supports the importance of follow-up. All other complications could be resolved and were not persisting. Furthermore, patients selected for inclusion in this study were optimal candidates for dental implants. Though the inclusion criteria predestinate the patient selection to some extent, the clinician’s expertise likely influences selection of a “good” patient. The patients included in our study had good overall health; American Society of Anesthesiologists (ASA) scores of 1 were observed for 85.6% of patients, and 74% of patients had never smoked.

A limitation of this study was the imbalance in the use of platform-matching and platform-switching abutments. Platform-switching abutments are relatively new, and in practice, the “newer” method (platform switching) was likely chosen over the conventional method. Platform-switching implants have been shown to have better outcomes with regards to bone level changes, but overall patient satisfaction does not differ between the two types [23], also supported by the results of our study. Another limitation may be the non-homogeneous study population. There were no exclusion criteria apart from the standard contraindications for an implant treatment, and the patients descend from the standard pool of private practices. Nevertheless, the success and survival rates were very high and were comparable with clinical data obtained in well-controlled clinical trials with multiple inclusion and exclusion criteria.

Within the limitations of this study, we conclude that the CAMLOG SCREW-LINE implants placed with both platform-matching and platform-switching abutments in patients in a private practice setting seem to achieve clinical outcomes comparable with those achieved in controlled clinical trials. The crestal bone changes over a 5-year period were mainly limited to < 1 mm and could be interpreted as proper peri-implant tissue stability. We also draw attention to the importance of patient education and regular follow-up on clinical outcomes. The patients in our study were highly satisfied with their implants, soft tissue parameters were excellent, and bone level changes were minimal, leading to good overall success and survival of the implants.

## Abbreviations

ASA: American Society of Anesthesiologists
MPI: Modified Plaque Index
PPD: Pocket probing depth
RCT: Randomized controlled clinical trial
SBI: Sulcus Bleeding Index
SD: Standard deviation
SLA: Sand-blasted, large grit, acid-etched
